# Factors that affect mass transport from drug eluting stents into the artery wall

**DOI:** 10.1186/1475-925X-9-15

**Published:** 2010-03-09

**Authors:** Barry M O'Connell, Tim M McGloughlin, Michael T Walsh

**Affiliations:** 1 Centre for Applied Biomedical Engineering Research (CABER), Department of Mechanical and Aeronautical Engineering and the Materials and Surface Science Institute, University of Limerick, Limerick, Ireland

## Abstract

Coronary artery disease can be treated by implanting a stent into the blocked region of an artery, thus enabling blood perfusion to distal vessels. Minimally invasive procedures of this nature often result in damage to the arterial tissue culminating in the re-blocking of the vessel. In an effort to alleviate this phenomenon, known as restenosis, drug eluting stents were developed. They are similar in composition to a bare metal stent but encompass a coating with therapeutic agents designed to reduce the overly aggressive healing response that contributes to restenosis. There are many variables that can influence the effectiveness of these therapeutic drugs being transported from the stent coating to and within the artery wall, many of which have been analysed and documented by researchers. However, the physical deformation of the artery substructure due to stent expansion, and its influence on a drugs ability to diffuse evenly within the artery wall have been lacking in published work to date. The paper highlights previous approaches adopted by researchers and proposes the addition of porous artery wall deformation to increase model accuracy.

## Introduction

Atherosclerosis is a degenerative disease that affects coronary, carotid and other peripheral arteries in the body. Disease formation can occur as early as childhood with the development of fatty streaks within the artery wall. As the aging process progresses these fatty streaks accumulate becoming larger lipid deposits within the artery and are detrimental to the smooth operation of the vasculature. Occlusions ensuing from aggressive plaque progression can often culminate in an ischemic attack, such as an apoplectic attack or a myocardial infarction [1-3]. There are a number of interventional procedures available to the clinician but the successful emergence of drug eluting stents (DES) has seen them become the preferred choice, so much so that by the beginning of 2006 more than 8 out of 10 coronary stents were DES [4] at a cost of between $4 and $5 billion annually [5].

When used in conjunction with balloon angioplasty, a bare metal stent increases post procedural patency by acting as a scaffold for the artery wall. Stenting however causes arterial injury resulting in the restenosis of a large portion of stented arteries. In an attempt to reduce the number of patients that warranted re-interventional procedures, stents were coated with therapeutic agents to combat restenosis and became known as DES. Drug loaded polymer coatings on the stent surface treat the injuries inflicted on the artery wall upon stent implantation. Once these drugs are within the wall they act against the arteries natural healing mechanisms in order to reduce the possibility of a recurring blockage. Quite often, when a DES is implanted in an artery a portion of the drugs used to target restenosis are washed away in the blood stream. Therefore it is imperative to understand all of the mechanisms that influence drug transport from the device so that DES can be designed to optimise their capabilities. This study began by defining the current knowledge base pertaining to drug transport within the artery wall from DES. However, the fundamental aspect concerning the mechanical deformation of the porous artery wall, and its influence on drug concentrations within, had not been reported in computational DES studies to date.

## Coronary artery disease

Coronary artery disease (CAD) has been intrinsically linked to atherosclerosis since the early 20^th ^century [1,6-8] and refers to the localisation of disease within the coronary arteries. CAD is the foremost cause of mortality in the world's industrialised nations [2,8-13] and is responsible for approximately 700,000 deaths in the United States of America annually [2,10]. Regular exercise and a balanced diet have been shown to influence CAD development but it is the concentration of lipid rich cholesterol in the blood that is considered the most important factor [8,14-18].

There are a number of ways to treat CAD such as coronary artery bypass graft (CABG) surgery. Although the long term patency rates of these grafts are moderate, CABG surgery remained the gold standard in the treatment of CAD until 1977 when the first percutaneous transluminal coronary angioplasty (PTCA) surgery was performed [19,20]. It was discovered that a substantial percentage of patients, reported to be between 30 and 60%, experienced recurrent ischemia due to the re-blocking of the artery (restenosis) within 6 months after PTCA. Restenosis is attributed to the mechanical injury caused by over dilating a device within the vessel resulting in neointimal hyperplasia, elastic recoil and negative arterial remodelling [4,19-26]. The next major advance in the field of minimally invasive interventional cardiology came in the form of the coronary artery stent (CAS). CAS reduced failure rates to between 10 and 40% [4,7,9,13,19-23,25,27-31] because it virtually eliminates elastic recoil and negative remodelling of the artery [32,33].

### Restenosis and its mechanisms

Restenosis can best be described as an overly aggressive inflammatory healing response in the artery wall due to the mechanical injury inflicted by balloon expansion. It is quantified by the reduction of lumen size after an intravascular interventional procedure [34]. Inflation of a balloon catheter within a diseased artery pushes the plaque against the artery wall, forcing the artery to stretch and often results in the fracture of not only the plaque but in some cases can damage the artery wall also [32,35]. Similar damage ensues when a stent is used in conjunction with a balloon catheter, however a stent can cause excessive injury, increasing neointimal formation, by penetrating the media and in extreme cases going as deep as the adventitial layer of the artery [22,32].

The development of restenosis can be influenced by three processes after PTCA, namely elastic recoil, arterial negative remodelling and neointimal hyperplasia [7,19,21-23]. Restenosis in up to 60% of patients undergoing PTCA can be attributed to the extent of elastic recoil and arterial modelling. Elastic recoil can occur within 1 hour of PTCA and is attributed to the passive recoil of the elastic medial layer of the artery. Positive remodelling of the artery wall occurs naturally in atherosclerotic arteries as a compensatory response to plaque formation and limits the narrowing of the vessel lumen. There are contrasting opinions on the mechanisms behind arterial remodelling [19] but the negative remodelling of the artery demonstrated after PTCA has been shown to contribute to restenosis by narrowing the artery. Both elastic recoil and arterial modelling are believed to be virtually eliminated when angioplasty is used in conjunction with a stent. Neointimal hyperplasia occurs in response to arterial injury, whereby platelets and fibrin are deposited on the wound. Growth factors are consequently released from the platelets which can promote smooth muscle cell (SMC) migration from the media. The result of this is a build up of SMC, extracellular matrix (ECM) and macrophages at the injured site over several weeks. It is here that cellular division takes place which appears to be essential for the development of restenosis [4,19,22,24,32,33,36-39]. Reduction of SMC proliferation is of the utmost importance if the incidence of restenosis is to be reduced [18,21,22,27,37,40,41]. The second generation of stent, the DES family, was developed with a coating of anti-restenotic agents aimed at alleviating the initiation of SMC proliferation to the site of injury after stent implantation.

## Drug eluting stents

Long term clinical trial results for this technology are not widespread, although several studies have reported on the performance of these devices in coronary arteries in the short to medium term. Some trials have indicated that the incidence of myocardial infarction and death are lower when DES are used [42,43] while other studies suggest that DES and BMS perform similarly [44]. The contrasting outcomes from such trials raise questions regarding the effectiveness of DES which has resulted in an increased effort by researchers to develop a means of predicting the behaviour of these devices in the short to long term.

As the primary use of these devices to date has been in the coronary arteries, this application will form the focus of the information presented. Mechanically speaking, stents offer far superior structural support than PTCA but the adverse biological responses need to be understood in order to address the mechanisms of injury that lead to restenosis. As described above, it is generally accepted that one of the main causes of restenosis following bare metal stent (BMS) implantation is SMC proliferation and migration from the media to the injured site. Thus, the curtailment of this excess SMC proliferation has been identified as the focal point in combating restenosis [18,21,22,27,37,40,41]. Attempts at systemic drug delivery to inhibit restenosis after stenting failed because effective dosing levels had a toxic effect and could not be tolerated by the patients [22,45]. Therefore the concept of local drug delivery using a sent platform was developed in an attempt to redress this. The site specific local delivery of drugs from DES offers an advantage over systemic delivery because the drug is applied to the injured vessel at the exact location and time that damage occurs.

The anti-restenotic coating on a DES inhibits the formation of neointimal hyperplasia by suppression of the inflammatory reaction, platelet activation and SMC proliferation. Most of the drugs explored originally were used as agents for anti-transplant rejection or as immunosuppressive drugs [21,46]. In April 2003 the first DES to gain commercial approval from the Food and Drug Administration (FDA) in the United States was the Cypher™ stent, which was developed by Cordis Corporation (Miami, FL. USA) and used a drug called sirolimus. Boston Scientific's (Natik, MA. USA) Taxus™ family of stents were the second DES platform approved by the FDA in March of the following year. The drug employed on the Taxus stent was called paclitaxel [47]. These first generation DES had a profound effect on reducing restenosis rates compared to BMS models. Clinical trials carried out on the Cypher stent (SIRIUS-1) showed restenosis rates of 8.9% after 8 months compared to 36.6% for BMS in the same study. Likewise the TAXUS IV trials heralded a dramatic reduction in restenosis rates when compared to BMS after 9 months, 7.9% versus 26.6% respectively [47].

In 2008, results were published from the SPIRIT-III clinical trials comparing Abbott Vascular's next generation everolimus DES, the Xience-V, with Boston Scientific's Taxus Express2 paclitaxel DES. The trial suggested that angiographic in-segment late loss was significantly less for the Xience-V stent than for the Taxus Stent. The trial also showed a dramatic reduction in major adverse cardiac events for the Xience-V stent compared to the Taxus stent, recording a 43.2% and 41.7% reduction after 9 and 12 months respectively [30]. Reasons for this may be the allocation of drug type or the design of the stent and its impact on the artery wall.

### Arterial mass transport

Mass transport (MT) refers to the transfer of mass, i.e. the species of interest which is drugs in the case of DES, from regions of high concentration to low concentration. In the absence of a free flowing system the presence of these concentration gradients induces diffusion, e.g. between the DES and the artery wall. MT can be broken up into two types within the human vasculature. Firstly blood side MT (BSMT) refers to species transport within the vessel lumen and is subject to the haemodynamics therein. The flowing nature of blood within the lumen will limit the DES ability to deliver therapeutic quantities of drug to the wall via BSMT, as it effectively washes the drugs away from the injured area. It is only in regions of high blood flow recirculation, e.g. immediately behind a stent strut, that it is possible for a sufficient amount of drug to remain for long enough to result in a therapeutic response.

The second mode is in relation to transport within the wall of the artery, referred to as wall side MT (WSMT). Along with the properties of the species being transported within the artery wall, WSMT depends on the structural condition of the wall itself, i.e. a damaged intimal layer could facilitate accelerated MT through to the medial layer [48]. The accurate modelling of MT within the artery wall requires a keen knowledge of the arterial substructure. Although a pressure induced velocity exists across a healthy artery wall, DES are implanted in arteries that are heavily blocked and in warrant of intervention. If the plaque were dense enough the transmural velocity would reduce to approximately zero. This would reduce the Peclet number and indicate a diffusion dominated mass transport environment within the artery wall.

**Figure 1:**
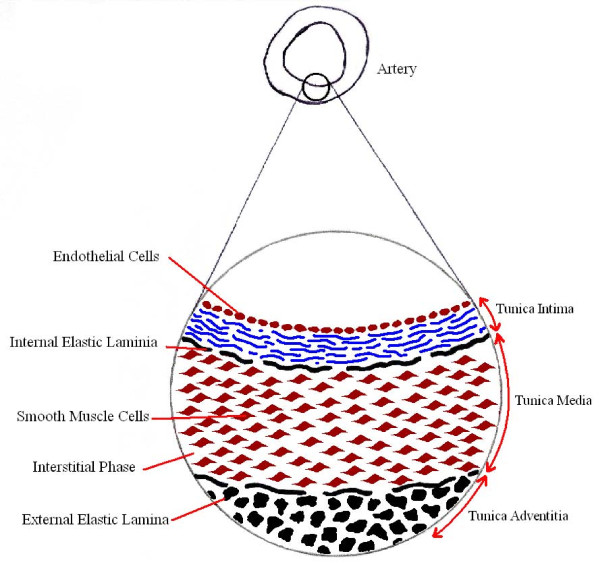
**Artery wall structure**.

The arteries themselves are not simply cylindrical mono-layered vessels. The wall consists of a complex multilayer porous substructure with the interstitial areas comprising predominantly of plasma. In a healthy artery this substructure (figure 1) is comprised of three concentric layers; the tunica intima, the tunica media and the tunica adventitia. The tunica intima is the innermost layer, comprising of a single layer of endothelial cells and a subendothelial layer mainly consisting of delicate connective tissues and collagen fibres. The outer boundary of the tunica intima is surrounded by an elastic tissue with fenestral pores known as the internal elastic lamina (IEL) [49]. The medial layer consists primarily of concentric sheets of SMC and elastic connective tissue. This configuration of SMC sheets enable the artery wall to contract and relax. The tunica media and the tunica adventitia are separated by another thin band of elastic fibres known as the external elastic lamina (EEL). The outermost layer of the artery, the tunica adventitia, comprises of connective tissue fibres and some capillaries. These fibres blend into the surrounding connective tissues and aid in stabilising the arteries within the body [17,49,50]. However, DES are deployed in arteries that have a significant amount of plaque which will undoubtedly have a bearing on arterial MT outcomes. The stenting procedure itself will also have an impact on MT due to the damage caused to the intimal layers upon stent deployment and subsequent thrombus build up around the stent. The greater the volume of clot that surrounds the strut, without being debilitating to blood flow, and the closer the strut is to the artery wall, provokes more optimal conditions for arterial MT.

Computational studies can give us vital information regarding the MT capabilities of a DES. Once the artery has been adequately defined changes can easily be made to computational models in order to assess their impact on the stents performance. Parameters such as the degree of artery wall damage and strut embedment, drug type, polymer characteristics and the extent and type of disease are documented in the following section. Although these studies give us an insight as to the behaviour of these variables, no published work to date contains a complete computational DES MT model because they all fail to incorporate the influence of the stent compressing the porous artery wall.

## Computational research

Spatial fluctuations in arterial drug concentration will depend on a host of variables such as the drug and polymer characteristics, stent design and also the structure of the artery wall and the plaque encased therein. Computational prediction of drug mass transport from a DES is a powerful means of evaluating the effectiveness of stent design before a stent is taken to clinical trial. This provides the manufacturer with a level of controllability, enabling the production of a DES that will offer sufficient dosage of therapeutic agents to the injured vessel while reducing the possibility of toxicity in locations of greater stent arterial coverage, e.g. at strut junctions.

The theoretical description and subsequent computational representation of mass transport from a DES into the artery wall presents significant challenges to researchers due to the complex interactions of all the physical and chemical processes taking place. Therefore it is necessary to make simplifications and assumptions in order to represent arterial MT and solve the problem computationally. One such simplification is to neglect the intimal layers of the artery, although they are known to effect species uptake within the wall. They are often neglected under the assumption that they become denuded upon stent implantation, leaving the stent strut in direct contact with the medial layer. This assumption is commonplace because it is the drug distribution within the medial layer that is ultimately responsible for reducing restenosis. From a computational perspective one of the more common ways to reduce the demand on numerical solvers is to decrease the degrees of dimensionality of the system from 3-D to 2-D and finally a 1-D analysis if need be.

In 2008, Zunino et al. [51] developed a comprehensive representation of a DES within an artery. The computational model was 3-D in nature and analysed the stent from a mechanical (upon balloon expansion) to a fluid dynamic (blood flow over the stent struts) perspective. A number of stent strut cells were modelled in contact with the artery and the concentration distribution was analysed. Reducing a models dimensionality saves on computational demands but not necessarily at the expense of realistic representation. Balakrishnan et al. published work [52-54] which demonstrated the progression of their 2-D computational DES transport model. The 2-D computational model presented in their most recent publication [52] may be perceived to be more realistic than the 3-D one proposed by Zunino et al. [51] due to the consideration of mural thrombus surrounding the stent struts. Likewise 1-D MT analysis can be effective in quantifying drug concentrations within the wall. Although dimensionally inferior, the work of Pontrelli and de Monte [55] describe MT through multiple layers, a porous artery medial layer and a porous polymer coating with polymer top coat (similar in composition to the Cypher stent [56]). The application of a porous artery wall subdomain is more representative of an actual artery wall than the homogeneous walls modelled in the aforementioned 2-D and 3-D examples.

### Influence of drug type on mass transfer

The hydrophobicity of drugs plays an important role in local drug concentrations from stents [48]. Hydrophilic drugs were found to wash away more quickly due to their tendency to follow transmural fluid movement, while hydrophobic drugs can attain higher concentrations in the artery wall due to their preferential binding to artery wall structural proteins. Hydrophilic drugs, such as heparin, were initially sought as the answer to combating restenosis but proved unsuccessful due to lower heterogeneous tissue concentrations than hydrophobic drugs. In contrast, drugs of a hydrophobic nature, such as paclitaxel and sirolimus, are advantageous due to their retention of high therapeutic doses within the wall [48]. Hwang et al. [48] suggest that diffusion in the media was anisotropic, with the circumferential diffusion being approximately 10 times greater in magnitude than the transmural value. In 2004, Zunino [57] conducted a numerical analysis comparing the employment of contrasting drug types, heparin and taxus. Heparin diffusivity within the wall was almost 3 times that of taxus. It was found that taxus accumulated more easily than heparin in the region surrounding the strut because of its tendency to bind with the tissue and its lower diffusivity. Zunino [57] recorded a 70% loss of heparin into the blood after 6 hours while for the same time period only 60% was lost for the taxus simulation.

Instead of attributing drug specific diffusion values in their analysis some researchers, such as Mongrain et al. [31], describe the concentration distribution in the artery wall for a range of arbitrary diffusion values. Their results indicate that the amount of drugs that accumulate within the artery wall depend on the values of the diffusion coefficient in the wall and the polymer respectively. Similar to Zunino [57], Mongrain et al. [31] deduced that drug accumulation within the wall was greater for smaller artery wall diffusivities as this reduces the drugs ability to leave the artery wall once embedded.

### Influence of strut positioning on mass transport

Strut positioning within an artery will ultimately be responsible for local fluctuations in arterial drug concentration. Unlike some of the first generation stents, with their uniformly distributed strut arrangement, the newer stents have irregular cell designs that enable greater flexibility while retaining structural stability. This generally results in an inhomogeneous distribution of stent struts in the circumferential and longitudinal directions. Hwang et al. [48] examined the effect of strut placement on a circumferential artery cross section (comparing 8 evenly with 8 randomly placed struts). Although mean tissue concentration remained the same regardless of strut position, the local concentrations, which predominantly determine biological response, are heavily dependent on strut spacing. Balakrishnan et al. [54] investigated the influences of multiple struts, strut spacing and the incidence of overlapping stent struts in a 2-D longitudinal artery cross section. A concentration of unity was placed on the stent surface and no polymer was used, although this is not ideal it does give a good approximation of the effect of the aforementioned variables after stent implantation. The highest wall concentration for longitudinal strut distribution was witnessed where the struts were closest together (1 strut length spacing apart), while a gap of 7 strut lengths resulted in individual peaks in concentration under each strut that increased slightly along the length of the model. For the case of overlapping struts, twice as much drug is available in close proximity and the flow around the struts is altered. Balakrishnan et al. [54] discovered that peak concentrations resulting from different overlapping strut configurations rise by 22-34% compared to the single strut case. The incidence of overlapping struts where one of them was fully embedded within the wall resulted in an increase of 45% in peak concentration. Mongrain et al. [31,58] conducted more thorough research relating to strut embedment and its effect on concentration within the wall. Degree of strut embedment was found to play a significant role in arterial concentration within the first 3 days of deployment. At 250 μm beneath the endothelium significantly higher concentrations were witnessed directly beneath any single strut compared to levels measured half way between two struts at the same depth. The study showed that after 3 days the wall concentrations normalised at both points due to the reduction of drugs being released from the polymer coating [31].

### Influence of polymer type on mass transport

Stent coatings play a vital role in the regulation of drug release from within. Careful consideration must be observed when allocating a polymer as a drug carrier for a DES. In this study the design of polymer coatings is not discussed in detail as these designs will ultimately only contribute to the diffusivity value of the drugs from the device. The following section reviews how varying polymer drug diffusivity (a consequence of polymer coating design) affects mass transport. If the polymer is not biocompatible an inflammatory response ensues [22,46] and in certain cases the development of intimal hyperplasia can be doubled when uncompatable polymers are used compared to a controlled substance [23]. Each biologically viable polymer must also be able to endure the stresses exerted with stent deployment in the arteries and must be able to resist cracking and peeling. They also have to maintain their physiochemical properties after sterilisation. There have been numerous polymers suggested as possible suitors for storing drugs on a DES but most of the polymers actually used on commercial DES are proprietary. Drug release rates can be altered with the addition of an extra layer of covering polymer to modulate between slow and fast release formulations. This adds an extra degree of complexity when designing functional DES.

Mongrain et al. [31,58] analysed how drug diffusivities within the polymer coating can impact drug concentration within the artery wall. While lower effective drug diffusivity values in the artery wall are favourable for uptake within the wall, having a low drug diffusivity in the polymer can be just as influential on the outcome. After 3 days an arterial concentration of 30.51% was achieved when the drug diffusivity in the polymer (D_polymer_) was 1 × 10^-14^m^2^/s but when D_polymer _was increased to 1 × 10^-12^m^2^/s the arterial concentration reduced to 3.55% (in both cases D_wall _was 1 × 10^-14^m^2^/s). Balakrishnan et al. [53] reiterates this finding but there are optimal polymer diffusivities that promote favourable arterial drug concentrations. At the extremes of drug diffusivity in the polymer coating, drug can be released so rapidly that it exceeds tissue absorption rate and the majority of drug can be lost to the blood stream. On the other hand if the drug is released too slowly it will not effectively penetrate the artery wall at levels sufficient to be therapeutic. The influence of dose and polymer thicknesses on the drug distribution within the wall was demonstrated by Balakrishnan et al. [53]. Drug release from thinner coatings with high concentrations was predicted to be fast initially but subject to a rapid decline when compared to a thicker polymer coating with lower concentration.

**Figure 2:**
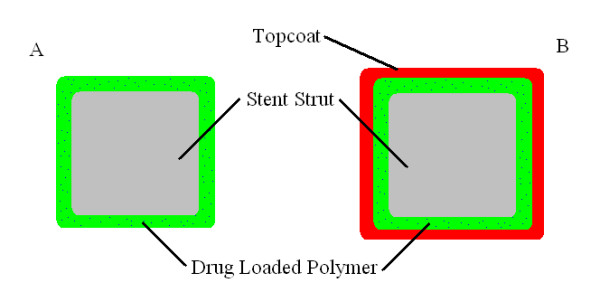
**Drug eluting stent strut cross-sections**. A) is a DES strut with a drug loaded polymer matrix and stent strut B) has a transport regulating topcoat.

Balakrishnan et al. [53] and Mongrain et al. [31,58] modelled their stent struts with a polymer matrix coating in which the drug is uniformly dispersed (figure 2A). The drug molecules further away from the surface have a longer migration distance, resulting in a lower drug release rate over time [56]. This type of configuration can be seen on Boston Scientific's Taxus™ stents. Another popular method of controlled release is a reservoir coating (figure 2B) as seen on Cypher™ stent developed by Cordis. In this configuration the stent is coated with a drug loaded base coat while a thin top coat acts as a rate controlling membrane [56]. Varying the diffusivity of the top coat regulates the transition of drug from the base coat to the artery wall.

### Influence of thrombus on mass transport

Hwang et al. [59] were among the first to explore the influence of thrombus height, width and type on the arterial drug uptake. Stents are often deployed at sites of thrombus and after implantation a clot will inevitably develop once the struts become covered with plasma proteins. In most cases this will not be angiographically present or clinically evident but even a fine layer of clotting blood deposited on the surface of the DES can alter drug distribution within the wall. The presence of clot alters the local environment of the stent strut and the physiological transport forces that regulate arterial uptake and retention. Late stent thrombosis has been reported to occur at a rate of 0.6% each year after DES implantation for up to 3 years [52] and strut position within any clot will have a major influence on the arterial uptake. The greater the mass of clot over the strut and the closer the strut is to the wall tends to provoke more optimal conditions for drug delivery. Hwang et al. [59] discovered that in this configuration concentration distribution in the wall can be 30 fold higher than situations where no clot is present. Similarly thrombus clots between the strut and the artery wall act as a buffer layer and reduce wall concentrations. Clot diffusivities higher than that of the arterial tissue can result in drug transfer from the stent to the artery wall at a rate faster than the wall can absorb. This results in the undesirable loss of drug to the circulatory system. Clots with diffusivities equal to or lower than the arterial wall transport drugs to the wall at a rate where the wall can effectively absorb the drugs, thus reducing drug loss to the bloodstream.

In 2008, Balakrishnan et al. [52] assessed how variations in mural thrombus size and distribution contribute to fluctuating arterial drug intensities. Their simulations indicate that thrombus cannot influence the slow rate of drug release from the stent because the polymer resistance to drug transport is significantly greater than that of the thrombus, which is consistent with *in vivo *experimental drug release. Local thrombus, just covering the strut, of 0.1 mm^2 ^increased peak average drug concentration and cumulative exposure by 80%. Greater clot formation between stent struts will also have an effect on arterial concentrations because once the species is transported within the clot it effectively increases the surface area from which the artery wall can absorb drugs. The formation of this interstrut thrombus, described as diffuse thrombus, acts as a shield from drug washout and culminates in an increase in arterial concentration by up to 3.5-fold [52].

The variability of thrombus can have a major impact on arterial drug concentrations. It can aid in drug uptake and retention within the wall when it covers the stent but too much thrombus will effectively block the artery, thus creating a problem that the DES aimed to alleviate. Also it can act as a barrier in preventing drugs from reaching the wall when it is located between the stent strut and the wall. The likely scenario following DES implantation within the vasculature is that at some location along the length of the stent each of these situations will be present. A computational approach considering variations in thrombus formation along the length of a stent would provide insight into resulting variations in arterial drug concentrations therein.

## Porous media

Drug transport within the vasculature is predominantly dependent on diffusion and convection. The haemodynamic nature of blood within the lumen results in convection dominated transport, while MT within the porous wall is heavily reliant on diffusion. The ratio of convective to diffusive forces is calculated by the Peclet number, *Pe = vL/D*, where *v*, *L *and *D *are the fluid velocity, characteristic length and species diffusivity respectively. Physiological Peclet numbers range typically from 0.1 to 10. A small *Pe *(i.e. *Pe *<< 1) is representative of transport which is dominated by diffusion, while a higher *Pe *(i.e. *Pe *>> 1) indicates convection dominated MT [50].

MT within the artery wall was studied in detail prior to the advent of stent based drug delivery devices in 2003. In 2002, Stangeby and Ethier [6] analysed the transport of macromolecules, such as low density lipoproteins (LDL), from the lumen through the endothelium and into the wall of a stenosed artery section. Subsequent research from Yang and Vafai [60] examined the effects of hypertension on LDL transport across each layer of the artery wall. In 2004, Tada and Tarbell [61] investigated the effects that the IEL has on macromolecule transport through the wall. Tada and Tarbell [61] took a more microscopic perspective of the artery wall than the others by modelling individual SMC. Although each of these studies differ somewhat, one thing they have in common is that the artery wall is treated as a porous structure. This approach may be applicable in cases of MT from a DES. DES are deployed in diseased arteries, a result of which is severely compromised artery wall filtration velocities. The resulting decrease in the Peclet number will reveal that MT is diffusion dominated in the artery wall. That is not to say the porous classification of the artery wall is no longer relevant when analysing MT from a DES. In fact the porous nature of any material, biological or otherwise, will ultimately determine the diffusion rates within the structure. Therefore any change in the structure of a porous media will alter its diffusion characteristics, as is the case for a stent expanding against an artery wall.

In previous computational studies that analyse the MT of drugs from stents only a few treat the artery wall as porous [48,55,57]. In all published research to date, to the best of the researchers' knowledge, the change in the structure of the artery wall under stent compression and its subsequent effect on drug MT have not been modelled computationally. However O'Connell and Walsh [62] have demonstrated this effect in an analogous porous material, which indicates that artery wall compression should be present in all DES computational models. Stent strut thickness may be vital in minimising the extent of arterial compression after DES expansion. The results from the recent SPIRIT-III clinical trials [30] could be an indication of the influence of strut thickness on arterial drug distribution and hence the ability to combat restenosis. It may be possible to develop a computational drug transport model based on these findings, thus creating a DES for optimal arterial MT. A reported difference of 60 μm in strut thickness between two different stents used in the SPIRIT-III trial may not necessarily result in 60 μm further compression due to the elastic nature of the artery. However an exploration into the extent of compression on the artery wall due to stenting would exhibit changes in arterial diffusivity compared to the case of a relaxed artery [62]. This can be attributed to a reduction in porosity coupled with an increase in tortuosity (equation 1) within the artery wall beneath the stent strut. The result of this relationship, described in equation 2, is an overall reduction in the effective diffusivity of drugs within the artery wall.

### Structural influence of arteries on mass transport

The structure of an artery is unique in that it enables uninterrupted blood flow throughout the body, providing the nutrients required for everyday function. For example its elastic nature enables it to contract and expand under pulsatile conditions induced from a beating heart. Along with a healthy endothelial layer to regulate species progression to the wall, the transport of such species once within the wall is influenced by the structure therein. A change in this structure will subsequently alter how the species are transported. When a stent is implanted within an artery it has a major impact on the structure of the wall. Differences in stent design will contribute to this because under similar balloon expansion criteria a thicker stent strut will result in greater compression of the artery wall. In the SPIRIT-III clinical trials, the Xience-V stent is 60 μm thinner than its Taxus counterpart and could be a factor behind its clinical success thus far. The thinner struts would cause less compression on the artery wall which in turn would better aid drug MT. Considering that the medial layer of the artery wall is only approximately 200 μm thick, any reduction in changes to its integrity could yield positive results. However greater radiopacity and structural support is achieved with a thicker strut [63].

Arterial properties such as permeability (*K*), porosity (*ε*), tortuosity (*τ*) and diffusivity (*D*) dictate the transport of drugs within the respective wall layers. The compression of these layers will alter these properties which in turn may inhibit the transport of species as governed by the MT equations. The permeability of a material is essentially its ability to permit the transfer of fluid through it. Permeability ultimately depends on the porosity and tortuosity of the artery wall and reducing it through wall compression following stent implantation compromises the arteries' ability to permit transport of drug.

**Figure 3:**
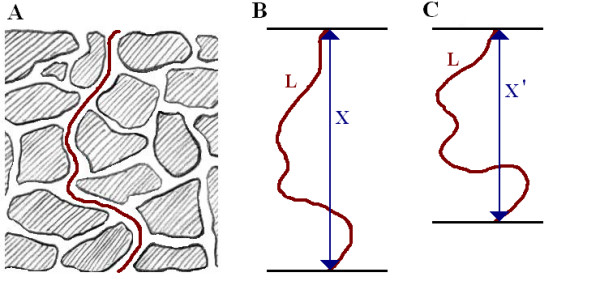
**Determination of tortuosity through a porous material using the arc-chord ratio**. The tortuosity of a path through a porous structure (A) can be determined by the ratio of the pore length, L, to the displacement, X. As can be seen in (B) and (C), the magnitude of L remains constant but as the compression increases the displacement X reduces to X' which results in an increase in tortuosity.

Porosity (*ε*) is a dimensionless parameter and is the ratio of pore volume to the total material volume. Any porous material under compression will demonstrate a smaller pore ratio than that of its relaxed state. This in turn influences the tortuosity (*τ*) as described by the pore path through the material. As the tortuosity increases so too does the effective distance over which diffusion has to take place [64]. It can be estimated by the arc-chord ratio, equation 1, which is the ratio of the actual pore length (*L*) to the distance between its ends (*X*), figure 3.(1)

The structure of the artery wall is highly heterogeneous and different layers will have different diffusivities. But the heterogeneity of these layers is highly regular and as such can be lumped into an effective diffusion parameter that characterises bulk drug transport within each respective artery layer [65]. Diffusion is caused by random molecular motion that eventually leads to complete mixing without the interaction of external forces. However, in a porous media diffusion takes place in confined tortuous pores and its progression is impeded as the tortuosity increases while the porosity decreases. The effect of pore size coupled with the tortuous nature of the arterial layers results in an effective diffusion (*D*_*eff*_) coefficient individual to each of its constituent layers and can be represented by equation 2 [64].(2)

where *D*_*Free *_is the free diffusivity and can be described by the Stokes-Einstein equation (3). This relationship uses molecule size to determine diffusivity.(3)

where *k *is the Boltzmann constant, *T *is body temperature, *μ *is fluid viscosity and *R *is the hydrodynamic molecule radius.

## Conclusions and future work

The shortcomings from PTCA and BMS procedures have been well documented since the devices became a prominent treatment of choice for interventional cardiologists in patients with severely stenosed coronary arteries. The underlying pathology of events such as restenosis have been investigated and it is widely accepted that proliferation of SMC from the medial layer of the artery to the site of injury is the primary cause of recurring ischemic events. Restenosis is regarded as a catastrophic failure for cardiovascular devices such as stents as it increases cost and trauma to the patient and can cause death if left untreated. The application of anti-proliferative agents on the surface of the stent introduced a greater degree of control that had been lacking in the first generation BMS. Quantification and associated predictive modelling of drug distribution from a DES in order to better understand the influence of arterial variables on MT is essential. This can only be achieved with realistic experimental validation of mass transport within a porous media for a range of compressive values. A more comprehensive understanding of the influences of such variables could lead to an optimal DES design which could provide a uniform distribution of drugs within the artery for a patient specific stenosis.

The therapeutic potential of a DES rests with its ability to distribute drugs evenly within the artery. Histological studies of excised stented arteries clearly depict arterial compression beneath stent struts [38]. Promising results from the thinner strut Xience-V stent in the SPIRIT-III clinical trials may explain the extent to which a decrease in strut thickness can have a positive effect on the clinical outcome. Knowledge of initial diffusivities for each of the constituent media is not enough when developing a predictive MT model of DES within a diseased artery. An iterative method taking into account the effect of stent strut compression on arterial diffusivity is required if a comprehensive understanding is to be achieved. Such improved numerical models of drug diffusion in arteries will lead to the development of favourable deployment conditions, responsible for the amount of artery wall compression, along with the development of desired drug properties which would enable the DES to deliver therapeutic quantities of drug to the artery wall as efficiently as possible.

It is evident that DES have made a major contribution to minimally invasive interventional cardiology by significantly reducing restenosis rates. A lack of knowledge regarding the mechanisms of drug transfer from these devices once implanted led DES developers to take a top-down approach to treating the problem. The stents were coated with 'enough' toxic drugs to combat restenosis and would give a beneficial response. This method had a clear commercial advantage over a slower bottom-up stent design approach, where all factors influencing MT would be assessed and taken into account during the DES design stage. Since their conception it has been the greater research community that has driven the increase in the knowledge base regarding MT from DES. This paper introduces the issue of compression mediating artery wall MT that has to date been overlooked in DES studies.

O'Connell and Walsh [62] examined this effect in an analogous experimental model and found a 35% decrease in effective diffusivity within the porous wall when it was compressed by 23.75%. Although their experiment was only a 1-D validation it gave insight into how compression can play an important role in regulating drug distribution within the artery wall. There are two main causes of concern associated with excessive artery wall compression. Firstly by reducing the artery wall diffusivity the drugs fail to distribute sufficiently throughout the artery wall, resulting in a concentrated dose of drugs beneath the stent strut. Secondly this excessive drug pooling can have a toxic effect on the artery wall. A study by Cho *et al*. in 2006 [66] reported on the ability of paclitaxel to inhibit human SMC proliferation with concentrations as low as 0.1 × 10^-3 ^mol m^-3 ^to a maximum of 1 × 10^-3 ^mol m^-3^. However, at the higher concentrations of paclitaxel a decrease in the proliferative activity of human endothelial cells was witnessed. Re-endothelialisation is a desired response after DES implantation and the pooling of drugs beneath the stent struts may adversely affect this process.

If a range of deployment conditions for a given stent design were known prior to implantation then a drug with a desired artery wall diffusivity could be allocated and loaded onto a polymer with the appropriate release characteristics. Likewise if a specific drug had to be delivered without compromising its diffusivity value then the stent design could be changed so as to minimise the damage/compression on the artery. As the hypothesis of compression mediating porous mass transport from DES is in its infancy there remains a substantial amount of work to first validate it in a 3-D arterial environment and subsequently model it computationally with the inclusion of the findings from previous studies. Ongoing studies to address these issues would ultimately result in the completion of a bottom-up understanding of the fundamentals of drug MT from a DES.

## Declaration of competing interests

The authors declare that they have no competing interests.

## Authors' contributions

BMOC analysed current literature and prepared the manuscript. TMM revised the manuscript, MTW supervised the study, revised and gave the final approval of the manuscript. All authors read and approved the final manuscript.
